# Impacts of ezetimibe on PCSK9 in rats: study on the expression in different organs and the potential mechanisms

**DOI:** 10.1186/s12967-015-0452-x

**Published:** 2015-03-14

**Authors:** Rui-Xia Xu, Jun Liu, Xiao-Lin Li, Sha Li, Yan Zhang, Yan-Jun Jia, Jing Sun, Jian-Jun Li

**Affiliations:** Division of Dyslipidemia, State Key Laboratory of Cardiovascular Disease, FuWai Hospital, National Center for Cardiovascular Disease, Chinese Academy of Medical Sciences and Peking Union Medical College, Beijing, 100037 China

**Keywords:** Ezetimibe, PCSK9, Liver, Intestine, Kidney, Molecular mechanism

## Abstract

**Background:**

Previous studies including our group have indicated the effects of ezetimibe on increased plasma proprotein convertase subtilisin/kexin type 9 (PCSK9) concentration, while the rapid expression in different organs and the potential molecular mechanisms for this impact have not been carefully evaluated.

**Methods:**

Thirty rats were randomly divided into two groups (n = 15 for each), which were orally administrated with ezetimibe (10 mg/kg/day) or normal saline. Blood samples were obtained at day 3 after orally administration, and the PCSK9 levels were determined by ELISA. We further analyzed the mRNA expression of PCSK9, low-density lipoprotein receptor (LDLR), sterol regulator element-binding protein 2 (SREBP2), and hepatocyte nuclear factor 1 alpha (HNF-1α) by real-time PCR, as well as the protein expression by western blot, in liver, intestine and kidney respectively.

**Results:**

Ezetimibe significantly increased plasma PCSK9 levels compared with control group, while there was no significant difference between the two groups with regard to lipid profile at day 3. Moreover, ezetimibe remarkably increased the expression of PCSK9, LDLR, SREBP2 and HNF-1α in liver. Enhanced expression of PCSK9, LDLR and SREBP2 protein were found in intestine and kidney, while no changes in the expression of HNF-1α were observed in intestine and kidney of rats with ezetimibe treatment.

**Conclusions:**

The data demonstrated that ezetimibe increased PCSK9 expression through the SREBP2 and HNF-1α pathways in different organs, subsequently resulting in elevated plasma PCSK9 levels prior to the alterations of lipid profile in rats.

## Introduction

Proprotein convertase subtilisin/kexin type 9 (PSCK9) is a circulating protein synthesized mainly in the liver, intestine, kidney and brain,which impairs low-density lipoprotein (LDL) clearance by binding directly to the epidermal growth factor repeat A of the LDL receptor (LDLR) and subsequently targeting it for degradation [1-4]. This process, subsequently, results in increased LDL-cholesterol (LDL-C) levels in the circulation [5,6]. Recent advances have indicated that statins (HMG-CoA redictase inhibitor) could eventually up-regulate PCSK9 levels in human by increasing the activity of sterol regulator element-binding protein 2 (SREBP2), which is a transcription factor that activates both LDLR and PCSK9 gene [7-10]. In addition, another common prescribed lipid-lowering drug, ezetimibe (an inhibitor of intestine cholesterol absorption) is widely used for the treatment of dyslipidemia, which could decrease LDL-C by approximately 20% when administered alone and when combined with statin could achieve 15% more decrease in LDL-C concentrations compared with statin alone [11]. However, limited data are currently available regarding the impact of ezetimibe on PCSK9 expression, especially in different candidate organs except for the liver. More importantly, the potential molecular pathways involved in PCSK9 expressions have less been investigated.

Our previous study showed that ezetimibe induced a rapid increase of plasma PCSK9 concentration prior to the decrease of LDL-C levels in rats, even after 3 days of administration [12]. Hence, the aim of the present study was to investigate (1) the rapid impacts of ezetimibe on PCSK9 expression in different organs; (2) the underlying mechanisms for the elevated plasma PCSK9 levels after ezetimibe treatment using this 3-days’ rat model.

## Materials and methods

### Materials

CircuLex rat PCSK9 enzyme-linked immunosorbent assay (ELISA) kit was purchased from CycLex Co., Nagano, Japan. The antibodies of PCSK9, LDLR and SREBP2 were purchased from Abcam Inc., Cambridge, MA, USA. The anti-hepatocyte nuclear factor-1 alpha (HNF-1α) was obtained from Santa Cruz Biotechnology Inc., Santa Cruz, CA, USA. Glyceraldehyde-3-phosphate dehydrogenase (GAPDH) and horseradish peroxidase-conjugated secondary antibody were purchased from Cell Signal Technology Inc., Beverly, MA, USA.

### Animal experimental design

Male Sprague–Dawley rats (weighing 180–220 g) were fed in a temperature-conditioned (22-24°C) room with alternating 12 h light/dark cycles and free access to food and water for 3 days to get familiar with the environment. At the start of the study, 30 rats were randomly assigned to two groups: (1) control group (n = 15, orally administrated with normal saline), (2) ezetimibe group (n = 15, orally administrated with ezetimibe 10 mg/kg/day). After 3 days’ orally administration, all rats were anaesthetized with pentobarbital sodium (40 mg/kg) via intraperitoneal injection and 2 ml fasting blood were collected from the inferior vena cava and transferred to K2 EDTA tubes. The blood samples were centrifuged, and the plasma was stored at −80°C until the analyses were performed. Immediately after blood sampling, livers, small intestines and kidneys were then removed and rapidly stored in liquid nitrogen for RNA and protein analysis. All experimental procedures and protocols were approved by the Care of Experimental Animals Committee of FuWai Hospital, Chinese Academy of Medical Sciences and Peking Union Medical College.

### Assay of plasma PCSK9 and lipid profile concentrations

Concentrations of total cholesterol (TC), triglycerides (TG), high density lipoprotein cholesterol (HDL-C) and LDL-C were determined on an automatic biochemistry analyzer (Hitachi 7150, Tokyo, Japan). Plasma PCSK9 concentrations were measured using a high sensitivity, quantitative sandwich enzyme immunoassay. The lower limit of detection was 0.038 ng/ml.

### Detection of PCSK9, LDLR, SREBP2 and HNF-1α mRNA expression in liver, intestine and kidney

Key enzymes of lipid metabolism, which were analyzed by real-time quantitative polymerase chain reaction (RT-PCR), were selected as candidate genes for the assessment of their mRNA expression levels in the liver, intestine and kidney of rats studied (Table 1). Briefly, total RNA in all tissue was isolated from hepatic tissue using the Trizol reagent Kit (Invitrogen, USA) following the manufacture’s instructions. And then, it was measured by spectrophotometry at an absorbance of 260 nm, and designated the purity valid if the ratio of A260/A280 was in the range from 1.8 to 2.0. The integrity of the RNA was checked by denaturing agarose gel electrophoresis and ethidium bromide staining. 3 μg of the total RNA was reversed transcribed by reverse Aid First Strand cDNA synthesis kit (Fermentas, CA, USA). The abundances of key genes (PCSK9, LDLR, SREBP-2 and HNF-1α) and GAPDH mRNA were analyzed by RT-PCR using the 7500HT RT-PCR system (Applied Biosystems, Foster, CA, USA). RT-PCR was performed using the SYBR Premix ExTaq (TaKaRa Bio Inc. Japan) according to the manufacturer’s instructions. Standard curves for each primer pair were generated by serial dilutions of cDNA from a reference sample and used for regression analyses. All PCR assays were performed in triplicate. The variance of the triplicate measurements was <1%. Results were analyzed using the standard curve method by the sequence detection systems software. The data was expressed as the relative levels of mRNA after normalized with GAPDH.

**Table 1:** **The sequences of primers for Real-Time PCR used in the study**

**Gene description**	**Primer**	**Sequences (from 5′ to 3′)**
PCSK9	F	GCTTCAGCGGCTTGTTCCT
	R	TGCTCCTCCACTCTCCACATAA
LDLR	F	CATCTTCCTCCCCATTGCA
	R	CCTCAGCCGCCAGTTCCT
SREBP2	F	AGCATACCGCAAGGTGTTCC
	R	CCAGGTGTCTACTTCTCCGTGT
HNF-1α	F	ATGACACGGATGACGATGGG
	R	ATGGGTCCTCCTGAAGAAGTGA
GAPDH	F	TGGCCTCCAAGGAGTAAGAAAC
	R	GGCCTCTCTCTTGCTCTCAGTATC

PCR: polymerase chain reaction; F: forward; R: reverse; LDLR: low density lipoprotein receptor; PCSK9: proprotein convertase subtilisin/kexin type 9; SREBP2: sterol regulatory element binding protein-2; HNF-1α: Hepatocyte Nuclear Factor 1 alpha; GAPDH: glyceraldehyde-phosphate dehydrogenase.

### Western blot analysis of PCSK9, LDLR, SREBP2 and HNF-1α protein expression

Western blot analysis of total protein was performed as previously described [13,14]. Approximately 150 mg of liver, small intestine and kidney tissue were used to extract protein for each assay respectively. Primary antibodies for anti-LDLR and anti-PCSK9 were used at a dilution of 1:1000, and primary antibodies for anti-SREBP2 and anti-HNF-1α were used at a dilution of 1:200. All secondary antibodies were used at a dilution of 1:2000. Protein bands were visualized by the ChemiDoc XRS system and the density was analyzed using the Quantity one software (Bio-Rad, USA).

### Statistical analysis

Data are expressed as mean ± standard deviation (SD). Comparisons between two groups were evaluated by using unpaired two-tailed *t*-test. A value of p < 0.05 was considered statistical significance. SPSS 19.0 statistical software package (SPSS Inc., Chicago, IL, USA) was used for all of the statistical analysis.

## Results

### Rapid effects of ezetimibe on plasma PCSK9 concentrations

**Figure 1:**
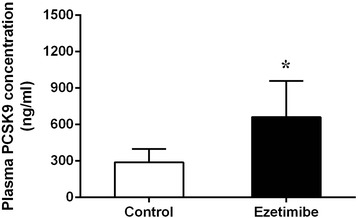
**The comparison of plasma PCSK9 levels in rats between control and ezetimibe groups.** *p < 0.05, compared with control group.

Table 1 showed the sequences of primers for Real-Time PCR used in the study. As shown in Table 2, there was no significant difference in lipid parameters between control and ezetimibe groups at day 3 (p > 0.05). While in rats receiving ezetimibe 10 mg/kg/day treatment for 3 days, plasma PCSK9 levels significantly increased compared with control group (661.37 ± 298.56 ng/ml vs. 289.36 ± 109.51 ng/ml, p < 0.05) (Table 2 and Figure 1).

**Table 2:** **The laboratory data of plasma PCSK9 levels and lipid profile in rats**

**Parameters**	**Group**	
	**Control (n = 15)**	**Ezetimibe (n = 15)**
PCSK9 (ng/ml)	289.36 ± 109.51	661.37 ± 298.56*
TG (mmol/L)	0.75 ± 0.68	0.59 ± 0.46
TC (mmol/L)	2.15 ± 0.23	2.13 ± 0.37
LDL-C (mmol/L)	0.54 ± 0.15	0.52 ± 0.13
HDL-C (mmol/L)	1.12 ± 0.30	1.21 ± 0.26

Data are expressed as mean ± SD. *p < 0.05, compared with control group.

*PCSK9*: proprotein convertase subtilisin/kexin type 9; *TG*: triglycerides; *TC*: total cholesterol; LDL-C: low density lipoprotein cholesterol; *HDL-C*: high density lipoprotein cholesterol.

### Changes of PCSK9, LDLR, SREBP2 and HNF-1α mRNA expression after ezetimibe treatment

**Figure 2:**
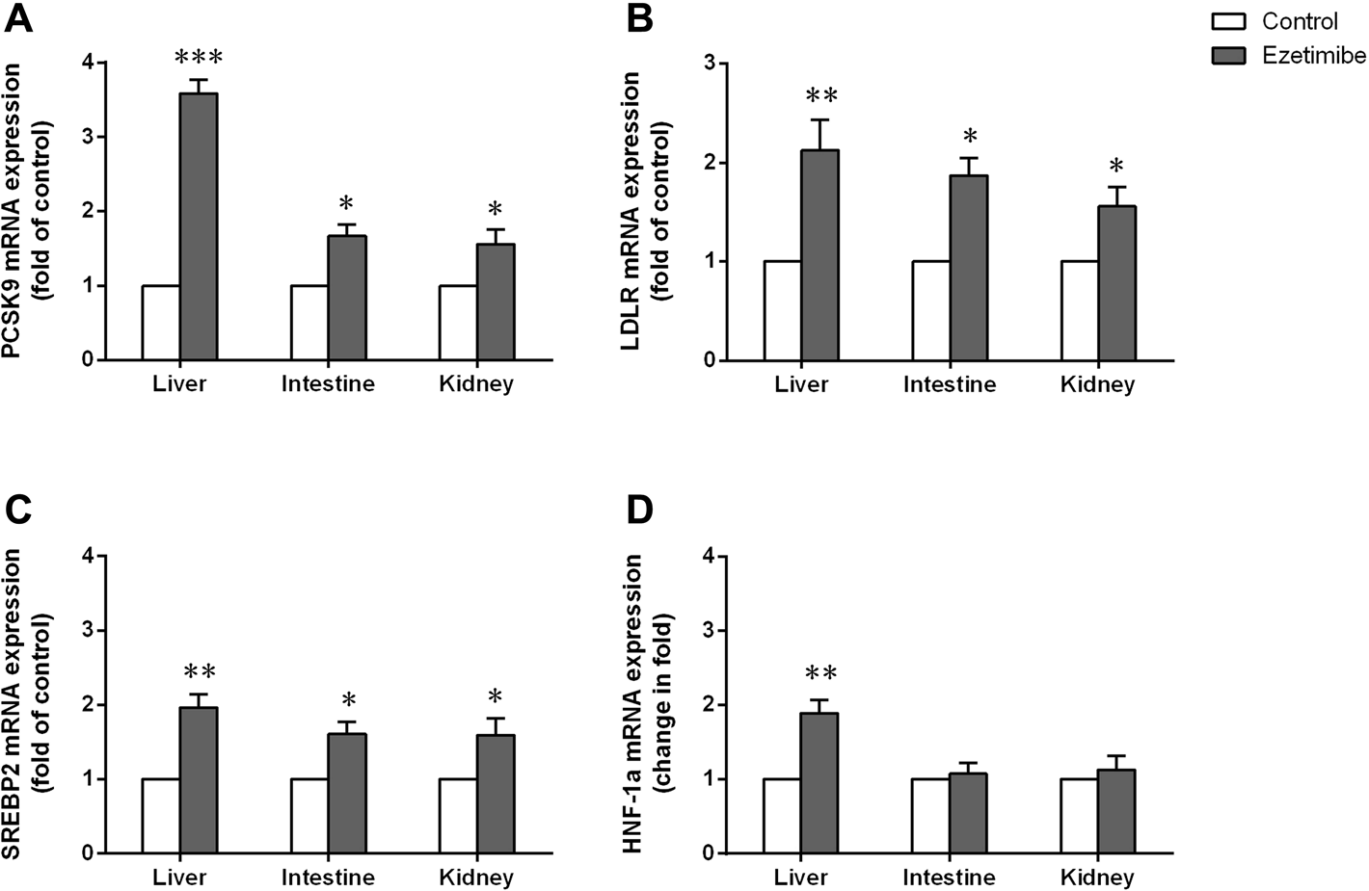
**The expression of PCSK9, LDLR, SREBP2 and HNF-1α mRNA analyzed by real-time PCR in liver, intestine and kidney respectively.** After ezetimibe treatment for 3 days, the mRNA expression of PCSK9 **(A)**, LDLR **(B)**, and SREBP2 **(C)** increased significantly in all organs compared with control group, while the expression of HNF-1α mRNA increased markedly only in liver **(D)**. Data are the mean ± SD. *p < 0.05, **p < 0.01, ***p < 0.001, compared with control group.

Relative mRNA expression of PCSK9, LDLR, SREBP2 and HNF-1α mRNA were determined in liver, intestine and kidney. As a result, the PCSK9, LDLR, SREBP2 and HNF-1α mRNA expression in liver of rats with ezetimibe treatment increased 3.6-fold, 2.1-fold, 2-fold and 1.9-fold respectively (p < 0.001 or p < 0.01, respectively) compared to the control group (Figure 2A-D). Moreover, the expression of PCSK9, LDLR and SREBP2 mRNA was significantly increased in both intestine and kidney after 3 days of ezetimibe treatment (all p < 0.05, Figure 2A-C). However, in the present study, no changes of the HNF-1α mRNA expression were found in intestine and kidney in ezetimibe group compared with the control group (all p > 0.05, Figure 2D).

### Effects of ezetimibe on PCSK9, LDLR, SREBP2 and HNF-1α protein expression

**Figure 3:**
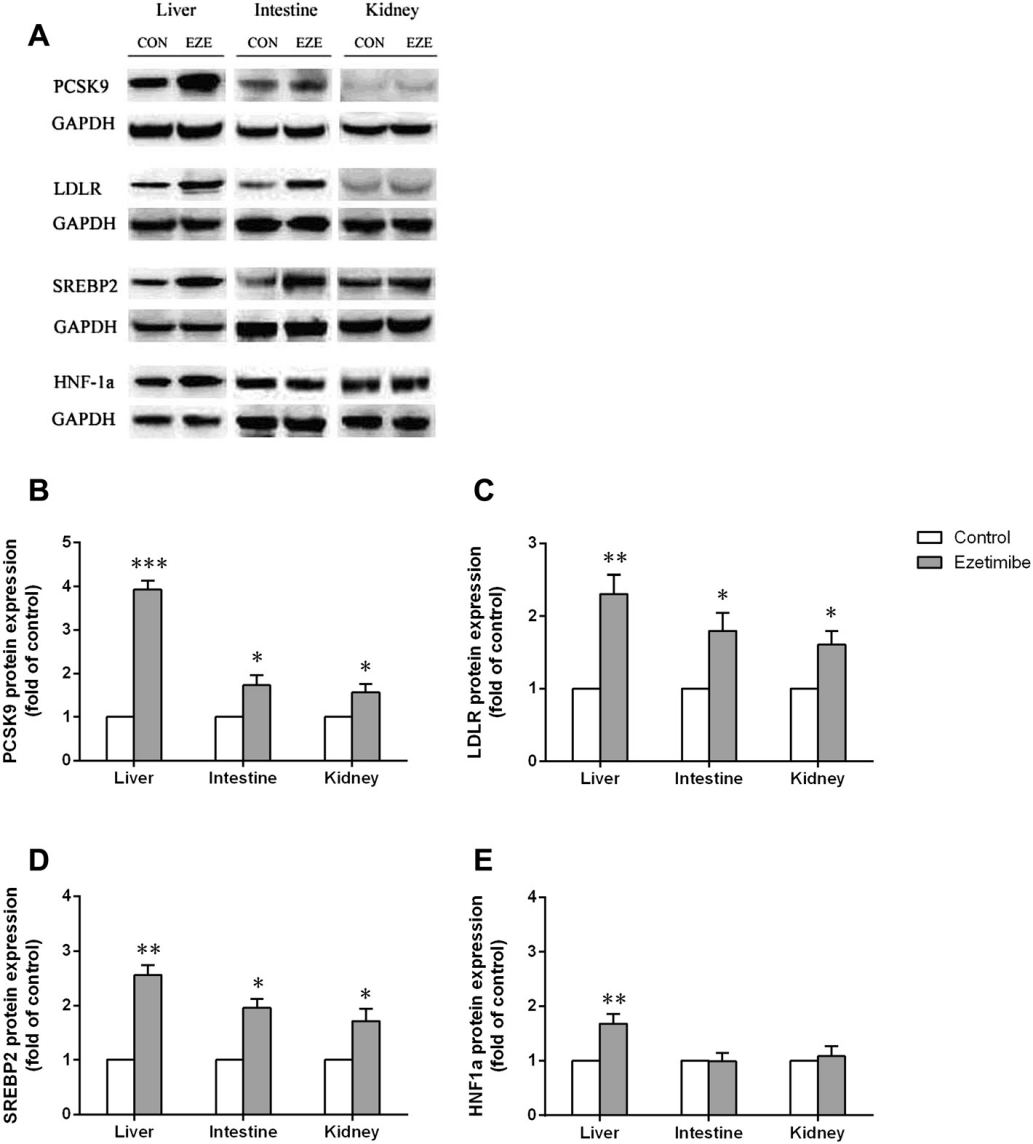
**The expression of PCSK9, LDLR, SREBP2 and HNF-1α proteins in liver, intestine and kidney respectively.** Western blot showed **(A)**: in ezetimibe group, the expression of PSCK9, LDLR and SREBP2 increased significantly in all organs,while the expression of HNF-1α increased markedly only in liver. Quantification analysis showed the expression of PCSK9 **(B)**, LDLR **(C)**, SREBP2 **(D)** and HNF-1α **(E)** in liver, intestine and kidney, respectively. CON: control; EZE: Ezetimibe. Data are the mean ± SD. *p < 0.05, **p < 0.01, ***p < 0.001, compared with control group.

The effects of ezetimibe 10 mg/kg/day treatment for 3 days on PCSK9, LDLR, SREBP2 and HNF-1α protein levels were also investigated in the present study. As shown in Figure 3, ezetimibe could remarkably enhance the protein expressions of PCSK9, LDLR, SREBP2 and HNF-1α in liver. Interestingly, the increased expression of PCSK9, LDLR and SREBP2 protein were found in intestine and kidney, while no changes in the expression of HNF-1α were observed in intestine and kidney of rats with ezetimibe treatment compared with the control group.

## Discussion

The impact of ezetimibe on circulating PCSK9 concentration and related mechanism has less been studied. In the present study, we confirmed that ezetimibe could rapidly increase the plasma PCSK9 concentration prior to the changes of lipid profile in rats. The main finding of this study is that ezetimibe could significantly increase the expression of PSCK9, LDLR, SREBP2 and HNF-1α in liver, intestine and kidney, but the expression of HNF-1α in liver alone in rats with ezetimibe 10 mg/kg/day after 3 days of treatment. Therefore, the present study may provide important novel findings including: 1) ezetimibe enhances the expression of PCSK9 through the multiple organ’s manner in rats; 2) SREBP2 pathway was involved in increased PCSK9 expression in all defined PCSK9-derived organs studied in rats, while HNF-1α pathway was a contributor for increased PCSK9 in liver alone.

PCSK9, which has a central role on cholesterol homeostasis by enhancing the endosomal and lysosomal degradation of hepatic LDLR, is closely related to lipid metabolism [15-17]. Therefore, PCSK9 has been considered as an important novel target for controlling dyslipidemia [18]. Previous studies have indicated that PCSK9 promotes the degradation of LDLR and can limit the beneficial effects of lipid-lowering drugs [19-21]. Multiple observations suggested that lipid-lowering drugs could elevate plasma PCSK9 concentrations [21,22] and might have rapid impact on PCSK9 levels [23]. Ezetimibe, an inhibitor of intestine cholesterol absorption, can decrease cholesterol synthesis and reduce circulating LDL-C concentration whenever it is prescribed alone or in combination with statins in controlling dyslipidemia [24,25]. Recently, several researchers have described that ezetimibe or the combination of ezetimibe and statin stimulates the expression of PCSK9 [12,26]. A study from Davignon J et al. reported that patients treated with statins alone had a 45% increase in PCSK9 levels and those treated with statin plus ezetimibe showed an approximately 77% increase in PCSK9 concentrations [27]. Hentze H et al. [28] indicated that plasma PCSK9 levels were increased by 137% in dyslipidemic cynomolgus monkeys after ezetimibe 14 days’ treatment accompanied with LDL-C decrease by 58%. A recent study from our group [12] demonstrated that the PCSK9 levels increased by 124% in rats administrated with ezetimibe 10 mg/kg/day for 3 days. Absolutely, these studies strongly suggested that ezetimibe could increase circulating PCSK9 concentrations in a rapid manner.

It has been reported that PCSK9 could be expressed in several organs, such as liver, intestine, brain and kidney [29-32]. Interestingly, Turpeinen H et al. [33] and Chen YQ et al. [34] reported that PCSK9 protein could be detected in coronary atherosclerotic plaque and even in cerebrospinal fluid. However, limited data are available regarding the impact of ezetimibe on PCSK9 expression, especially in different organs. Galman C et al. demonstrated that ezetimibe treatment reduced cholesterol absorption, while the expression of hepatic PCSK9 mRNA was unaltered in aged rats [35]. A recent study investigated the impacts of ezetimibe 10 mg per day and simvastatin 40 mg per day, alone and in combination (ezetimibe 10 mg per day plus simvastatin 40 mg per day) therapy for 14 days in 72 healthy men on PCSK9 using a single center, randomized, open-label design [36]. They found that ezetimibe did not increase circulating PCSK9 concentrations while simvastatin did, whenever ezetimibe was administered alone or in combination with simvastatin. Gouni-Berthold et al. [25] indicated that ezetimibe had no effect on the expression of PCSK9 and LDLR protein in mononuclear blood cells from healthy men. Therefore, further exploration of the effects of ezetimibe on PCSK9 and potential organs may be of great interest. Our study, for the first time, indicated that ezetimibe up-regulated PCSK9 mRNA and protein expressions in liver, intestine and kidney in rats receiving ezetimibe 10 mg/kg/day for 3 days. The disparity for their trial in humans and our study in rats regarding the effects of ezetimibe on plasma PCSK9 level may be due to the subjects studied.

In addition, limited published studies investigated potential mechanisms for the increased expression of PCSK9 stimulated by ezetimibe. Brandon Ason et al. [37] applied qRT-PCR to analyze the expression of 361 genes involved in hepatic lipid metabolism and found that many genes within the SREBP-2 pathway were induced following ezetimibe treatment (2.5-fold average induction relative to control). This study provides theoretical evidences about the impact of ezetimibe on plasma PCSK9 levels, that is, the enhanced PCSK9 expression may be also associated with SREBP-2 pathway. Several previous studies have indicated that the impact of statins on PCSK9 expression was mainly related to SREBP-2 pathway. Besides, Li H [38] et al. also showed that a coordinate reduction of nuclear SREBP2 and HNF-1α by berberine led to a strong suppression of PSCK9 transcription in HepG2 cells, indirectly indicating a regulation of SREBP2 and HNF-1α on PCSK9. In our study, data suggested that ezetimibe significantly increased hepatic SREBP2 and HNF-1α expression, and dramatically up-regulated the SREBP2 expression in intestine and kidney. However, there were no significant differences in HNF-1α expression in intestine and kidney of rats treated with ezetimibe compared to that of control rats. Additionally, the reports by Telford DE et al. [26] and Repa JJ et al. [39] demonstrated that ezetimibe could enhance the expression of hepatic LDLR mRNA which was increased by 1.7-fold in pigs and 2-fold in wild-type mice, respectively, through SREBP2 pathway activation. Consistent with previous studies, we found that ezetimibe markedly increased the expression of LDLR and SREBP2 in all organs of rats investigated in the present study. Additionally, both PCSK9 and LDLR gene transcription are under the control of SREBP2, which was activated by the low intracellular cholesterol levels. Moreover, the intracellular cholesterol levels were decreased more quickly than the plasma after the lipid-lowering drug treatment [10]. Therefore, in the present study, the SREBP2 pathway was dramatically stimulated even when the plasma cholesterol concentration was not changed. Nonetheless, further studies are needed to observe the long-term effect of ezetimibe on plasma PCSK9 and the role of different pathways of different organs in the regulations of circulating PCSK9.

## Conclusion

In this 3-days’ rat model with ezetimibe administration, our data demonstrated that ezetimibe could enhance PCSK9 expression rapidly, subsequently resulting in elevated plasma PCSK9 levels prior to the alterations of lipid profile, which was through the SREBP2 and HNF-1α pathways in different organs.
